# Fructose Consumption, Lipogenesis, and Non-Alcoholic Fatty Liver Disease

**DOI:** 10.3390/nu9090981

**Published:** 2017-09-06

**Authors:** Kasper W. ter Horst, Mireille J. Serlie

**Affiliations:** Department of Endocrinology and Metabolism, Academic Medical Center, Meibergdreef 9, 1105AZ Amsterdam, The Netherlands; k.w.terhorst@amc.nl

**Keywords:** fructose, hepatic steatosis, NAFLD, de novo lipogenesis, lipid synthesis, ChREBP, insulin resistance, obesity, metabolic syndrome

## Abstract

Increased fructose consumption has been suggested to contribute to non-alcoholic fatty liver disease (NAFLD), dyslipidemia, and insulin resistance, but a causal role of fructose in these metabolic diseases remains debated. Mechanistically, hepatic fructose metabolism yields precursors that can be used for gluconeogenesis and de novo lipogenesis (DNL). Fructose-derived precursors also act as nutritional regulators of the transcription factors, including ChREBP and SREBP1c, that regulate the expression of hepatic gluconeogenesis and DNL genes. In support of these mechanisms, fructose intake increases hepatic gluconeogenesis and DNL and raises plasma glucose and triglyceride levels in humans. However, epidemiological and fructose-intervention studies have had inconclusive results with respect to liver fat, and there is currently no good human evidence that fructose, when consumed in isocaloric amounts, causes more liver fat accumulation than other energy-dense nutrients. In this review, we aim to provide an overview of the seemingly contradicting literature on fructose and NAFLD. We outline fructose physiology, the mechanisms that link fructose to NAFLD, and the available evidence from human studies. From this framework, we conclude that the cellular mechanisms underlying hepatic fructose metabolism will likely reveal novel targets for the treatment of NAFLD, dyslipidemia, and hepatic insulin resistance. Finally, fructose-containing sugars are a major source of excess calories, suggesting that a reduction of their intake has potential for the prevention of NAFLD and other obesity-related diseases.

## 1. Introduction

Obesity and obesity-related cardiometabolic conditions are an epidemic threat to public health [1]. Globally, more than one in three adults is now overweight or obese, and the prevalence of overweight and obesity continues to rise in both developed and developing countries [2]. Obesity predisposes to several medical complications, including, but not limited to hypertension, dyslipidemia, non-alcoholic fatty liver disease (NAFLD), type 2 diabetes, atherosclerosis, and some forms of cancer [3]. As a consequence, overweight and obesity are associated with increased risks for all-cause and cause-specific mortality [4,5].

Often considered the hepatic manifestation of the metabolic syndrome, NAFLD is defined by hepatic steatosis (that is, intrahepatic lipid accumulation) in the absence of apparent liver disease or excessive alcohol intake [6]. Hepatic steatosis is commonly diagnosed when ≥5% of hepatocytes contain large lipid droplets or when intrahepatic triglyceride content is >5.6% [7,8], but note that the definition of normal liver fat content depends on the assessment method. In this regard, the magnetic resonance spectroscopy-based cutoff of 5.6% (that is, liver volume comprised of fat) corresponds to approximately 15% histologically confirmed steatosis (that is, hepatocytes with macrovesicular steatosis) [9]. Alarmingly, on the basis of a recent meta-analysis of 45 imaging studies, an estimated quarter of the global adult population suffers from NAFLD [10]. This is a cause for major concern, because NAFLD increases the risk for type 2 diabetes and cardiovascular disease [11]. Moreover, simple steatosis in the context of NAFLD may progress to non-alcoholic steatohepatitis (NASH) and liver fibrosis [12,13,14]. As such, NAFLD/NASH is now the third leading cause for liver transplantation [15,16].

Intrahepatic lipid accumulation may be the result of, in parts, increased delivery of fatty acids (FA) to the liver, increased hepatic FA synthesis from de novo lipogenesis (DNL), and/or decreased lipid clearance through secretion or oxidation [17,18,19]. Although the relative contribution of these pathways to the development of clinical NAFLD is only partially known, several human trials have demonstrated that increased DNL is a particularly important abnormality in NAFLD [18,19,20]. Overconsumption of added sugar, the intake of which has increased dramatically over the past century [20], is associated with obesity, NAFLD, and insulin resistance [21,22,23,24,25,26]. Some of these associations may be the result of stimulation of hepatic DNL by dietary sugars [27,28,29]. Notably, most added sugars, including sucrose and high-fructose corn syrup, are made up of near-equal shares glucose and fructose, but fructose is emerging as the potentially harmful component [29]. Unlike glucose, ingested fructose is preferentially metabolized by the liver [30]. This and several other features of fructose metabolism make it an exceptionally lipogenic sugar [20,29,31].

In this review, we will focus on the role of fructose consumption in the development of hepatic steatosis. To this end, we will first discuss fructose physiology and the molecular mechanisms that may link fructose to NAFLD and insulin resistance. We will then consider the available evidence from studies in humans to determine the impact of fructose metabolism on clinically relevant outcomes. Finally, we will use this framework to discuss opportunities for therapy. Our review methods are summarized in Appendix A. Recommendations for further reading are listed in Appendix B. 

## 2. Fructose Metabolism in Physiology

Food and drink contain fructose as monosaccharide (free fructose), disaccharide (sucrose), or polysaccharide (fructan). Whereas free fructose is absorbed directly from the intestinal lumen, fructose in larger carbohydrate molecules is first cleaved off at the lumenal membrane of enterocytes [32]. Intestinal fructose transport is mainly facilitated by glucose transporter 5 (GLUT5/SLC2A5) on the lumenal side and glucose transporter 2 (GLUT2/SLC2A2) on the basolateral side [33,34,35]. Once in the portal circulation, ingested fructose is transported into the liver via specific hexose transporters. Hepatic GLUT2 is thought to be the primary glucose and fructose transporter [35,36]; however, glucose transporter 8 (GLUT8/SLC2A8) has also been shown to mediate fructose transport into hepatocytes and hepatic fructose metabolism [37]. Other glucose transporter family members are likely involved as well [38]. Ingested fructose is almost completely extracted from the portal blood upon first-pass [30]. In contrast to ingested glucose, only a small fraction of ingested fructose will ultimately enter systemic circulation [39,40].

**Figure 1:**
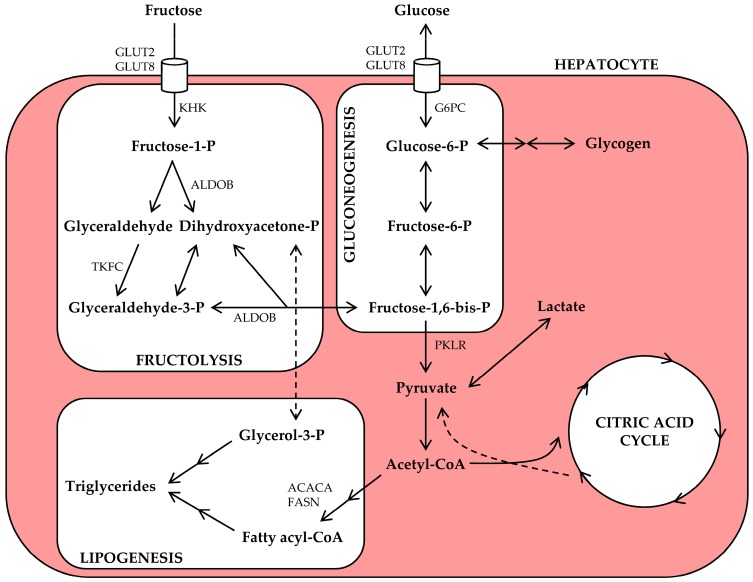
Hepatic fructose metabolism provides substrate for multiple metabolic pathways. Ingested fructose is transported from the portal vein into hepatocytes via glucose transporter 2 (GLUT2) and glucose transporter 8 (GLUT8). Ketohexokinase (KHK) converts intracellular fructose to fructose-1-phosphate (fructose-1-P), which is then split into glyceraldehyde and dihydroxyacetone-phosphate (dihydroxyacetone-P) by aldolase B (ALDOB). Glyceraldehyde is further phosphorylated to glyceraldehyde-3-phosphate (glyceraldehyde-3-P). Fructolysis-derived triose phosphates can now be used for gluconeogenesis, glycogen synthesis, lactate production, or acetyl-CoA production, and the latter can be oxidized or used for lipogenesis. Other abbreviations: ACACA, acetyl-CoA carboxylase α; FASN, fatty acid synthase; G6PC, glucose-6-phosphatase; P, phosphate; PKLR, pyruvate kinase; TKFC, triokinase.

An overview of hepatic fructose metabolism is presented in Figure 1. Briefly, hepatic fructolysis is initiated by phosphorylation of fructose into fructose-1-phosphate by ketohexokinase (KHK). Through aldolase B (ALDOB) and triokinase (TKFC) activity, fructose-1-phosphate is then split into two triose phosphate intermediaries. Fructolysis bypasses the rate-limiting steps of glycolysis and is therefore much faster; ingestion of fructose rapidly results in the availability of downstream (triose phosphate) intermediaries [30,41,42]. At this point, fructose and glucose metabolic pathways converge, and the triose phosphate intermediaries may now enter the gluconeogenesis, lipogenesis, or oxidation pathways [42].

Although hepatocytes possess the enzymatic machineries to convert fructose-derived carbon into glucose, glycogen, lactate, lipid, carbon dioxide, and/or other metabolites [42], the actual metabolic fate of ingested fructose likely depends on various factors, including nutritional status, long-term dietary patterns, and/or genetic make-up. Isotope-labeled metabolic tracer techniques offer attractive means to quantitatively evaluate the conversion and oxidation of ingested fructose, and several of such tracer studies have been performed in humans. After administration of an acute oral fructose load to healthy non-exercising subjects, most ingested fructose is converted to glucose (29–54%, part of which may then be incorporated into glycogen) or lactate (~28%). A small percentage (~1%) enters the DNL pathway for conversion into lipid [39]. Despite these estimated conversion rates, acute fructose ingestion produces only minor increases in plasma glucose and insulin levels [43], whereas it consistently raises plasma triglyceride levels [43,44,45,46,47]. Isotope tracer studies also show that 31–59% of ingested fructose is oxidized (and ultimately converted into carbon dioxide) within 3–6 h after ingestion [39]. Thus, the hypertriglyceridemic effect of an acute fructose dose may involve metabolic pathways other than DNL, including a shift towards carbohydrate oxidation, thereby sparing lipids [39], or a decrease in peripheral tissue lipid uptake [45]. Chronic fructose consumption induces additional changes that promote hepatic lipid synthesis, and this will be discussed below.

## 3. What Are the Mechanisms Linking Fructose to NAFLD?

**Figure 2:**
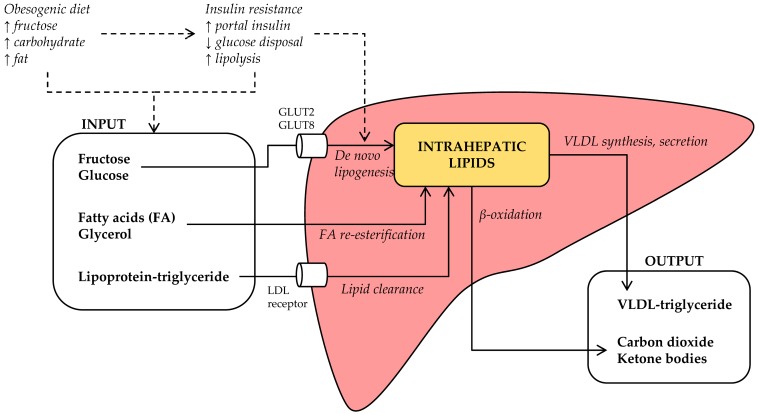
Hepatic steatosis develops when lipid input exceeds lipid output. Lipid input consists of (i) de novo lipogenesis from (primarily carbohydrate) precursors, (ii) re-esterification of lipolysis-derived fatty acids (FA) and glycerol, and (iii) low-density lipoprotein (LDL) receptor-mediated endocytosis of remnant-triglycerides. Lipid output consists of (i) β-oxidation and (ii) secretion of triglycerides in very low-density lipoprotein (VLDL) particles. Other abbreviation: GLUT2, glucose transporter 2; GLUT8, glucose transporter 8.

Intrahepatic lipids accumulate when the rate of hepatic lipid input exceeds the rate of hepatic lipid output (Figure 2). On the input side, lipids may be synthesized (de novo) from carbohydrates or other precursors, imported as lipolysis-derived FA, or imported as lipoprotein-derived triglyceride. Tracer experiments have demonstrated that all three input sources contribute significantly to intrahepatic lipid stores: ~59% of intrahepatic triglycerides in NAFLD patients derives from circulating FA, whereas ~26% derives from DNL and ~15% from dietary fat [18]. However, when NAFLD patients were compared to matched controls without steatosis, there was no meaningful difference in the FA flux from adipose tissue to liver [19]. In contrast, DNL was 3-fold greater in NAFLD compared to the control subjects [19], suggesting that increased DNL is a distinct pathophysiological feature of human NAFLD.

On the output side, intrahepatic lipids may be utilized in β-oxidation, leading to the production of carbon dioxide or ketone bodies, or secreted as triglyceride in very low-density lipoprotein (VLDL) particles. Defects in these pathways have also been demonstrated, although interpretation of these data seems more complicated. Under fasting conditions, hepatic lipid oxidation rates are unchanged in NAFLD patients [48]; however, high fructose consumption may promote a shift towards carbohydrate oxidation [39]. In addition, NAFLD was associated with increased hepatic VLDL secretion in some [49,50,51], but not in all trials [19], whereas NASH was associated with decreased VLDL production [52]. In the following sections, we will discuss if and how fructose consumption influences these mechanisms.

### 3.1. Fructose and Lipid Synthesis

**Figure 3:**
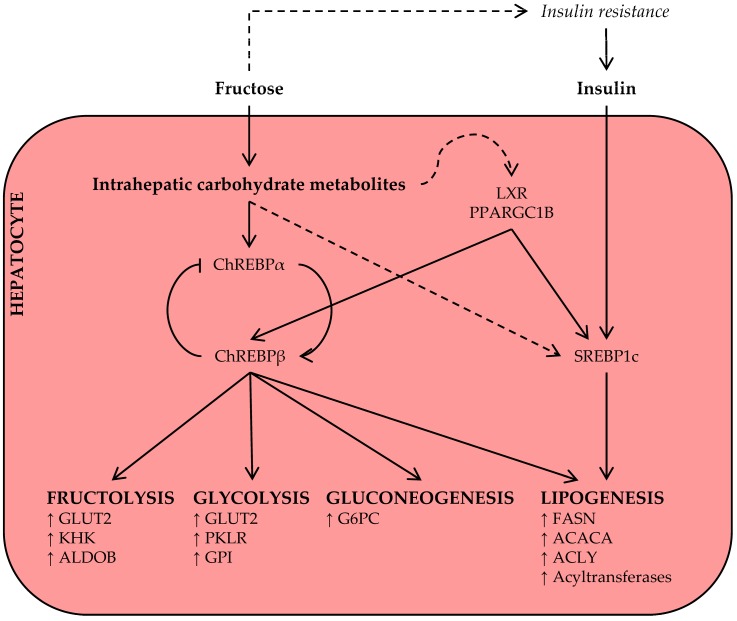
Chronic fructose consumption promotes mechanisms that upregulate the hepatic lipogenesis program. Hepatic metabolism of ingested fructose increases levels of intrahepatic carbohydrate metabolites. These act as nutritional regulators of key transcription factors, including carbohydrate response element-binding protein (ChREBP) and sterol regulatory element-binding protein 1c (SREBP1c), and coactivators for genes in multiple metabolic pathways. Stimulation of the lipogenesis program is further amplified by hyperinsulinemia in the context of insulin resistance. Other abbreviations: ACACA, acetyl-CoA carboxylase α; ACLY, ATP citrate lyase; ALDOB, aldolase B; FASN, fatty acid synthase; G6PC, glucose-6-phosphatase; GLUT2, glucose transporter 2; GPI, glucose-6-phosphate isomerase; KHK, ketohexokinase; LXR, liver X receptor; PKLR, pyruvate kinase; PPARGC1B, peroxisome proliferator-activated receptor γ coactivator 1β.

As discussed above, acute hepatic fructose metabolism rapidly results in the availability of DNL substrate, and some authors have suggested that this may largely drive the increase in plasma triglyceride levels after acute fructose ingestion [29]. This, however, has yet to be confirmed in human metabolic tracer studies [39]. The available evidence suggests that only a small percentage of fructose-derived carbon is directly converted into lipid within 4–6 h [45,53]. However, labeled acetate infusion studies do support DNL-promoting effects of fructose in the subacute-to-long term [46,54,55], suggesting that chronic fructose exposure induces prolipogenic mechanisms (Figure 3), in addition to increasing the availability of DNL substrate.

Carbohydrate response element-binding protein (ChREBP), also known as Mlx interacting protein-like (MLXIPL), is a key transcription factor for enzymes in the fructolysis, glycolysis, gluconeogenesis, and DNL pathways [56,57,58]. The least potent isoform, ChREBPα, is inhibited under conditions of low glucose [59]; however, upon activation by intracellular carbohydrate metabolites, ChREBPα induces the transcription of a more potent isoform, ChREBPβ, from an alternative promotor [60]. Thus, the ChREBPα/β mechanism senses intracellular carbohydrate signals and regulates the expression of metabolic gene programs in response to increased carbohydrate availability [61].

As fructose is primarily metabolized by the liver [30], fructose, more than glucose, may give rise to the intrahepatic carbohydrate metabolites that activate hepatic ChREBP independently of hepatic insulin signaling [29,62,63,64]. Observations in multiple species now support an important role for hepatic ChREBP in fructose metabolism and metabolic disease. In rats, high-fructose feeding, as compared to glucose, was associated with increased ChREBP activity and expression of its target genes [65]. In accordance, high-fructose feeding to mice increased hepatocellular carbohydrate metabolites, expression of ChREBP target genes, and hepatic steatosis, and these adverse metabolic effects of the high-fructose diet were fully dependent on hepatic ChREBP activation [58]. In another recent rodent study, fructose-activated ChREBP induced the hepatic expression and secretion of fibroblast growth factor 21 (FGF21), a response that was essential for adaptive fructose metabolism [66]. Fructose ingestion also acutely raised circulating FGF21 levels in humans [43,67], warranting further investigation into the ChREBP-FGF21 signaling axis in fructose metabolism. 

Notably, in the absence of ChREBP, high-fructose diets do not cause hepatic lipid accumulation, but inflammation and early signs of fibrosis instead [68]. It thus seems that ChREBP-mediated lipogenesis is required to prevent even more adverse hepatotoxicity upon fructose consumption, but that this adaptive mechanism may cause hepatic steatosis when too much fructose is consumed [69]. Increased hepatic ChREBP expression is also associated with NAFLD and insulin resistance in obese humans [70], but a direct effect of ingested fructose on ChREBP in human liver has not yet been demonstrated.

Sterol regulatory element-binding protein 1c (SREPB1c) is another key transcription factor for genes in the DNL pathway and implicated in the development of NAFLD [71]. The expression and post-translational activation of SREBP1c are strongly stimulated by insulin signaling; this mechanism ensures a coordinated DNL response under conditions of high glucose availability [57]. High-fructose diets commonly induce systemic insulin resistance and fasting hyperinsulinemia [58,72,73,74], thereby promoting insulin-mediated SREBP1c activation and hepatic lipid synthesis. However, the observations that insulin-depleted [75] and liver-specific insulin receptor knockout [76] mice also display an induction of SREBP1c upon acute sugar administration or on a short-term high-fructose diet indicate that this transcription factor is also regulated by nutrient signals, independent of insulin signaling. Finally, monosaccharides activate other transcriptional coactivators, including liver X receptor (LXR) [77] and peroxisome proliferator-activated receptor γ coactivator 1β (PPARGC1B) [78], that may amplify the ChREBP and SREBP1c-mediated lipogenesis response.

In summary, ingested fructose, unlike glucose, is primarily handled by hepatocytes, increasing the availability of intrahepatic carbohydrate metabolites. These provide hepatocytes with both DNL substrate and regulatory signals, mediated via several key lipogenesis transcription factors, for effective hepatic lipid synthesis.

### 3.2. Fructose and Lipolysis

In healthy human subjects, acute fructose ingestion is associated with a decrease in circulating FA levels [79], suggesting inhibition of adipose tissue lipolysis or increased FA clearance, but the signaling mechanism is currently unknown. An antilipolytic effect of fructose was also demonstrated in isolated rat adipocytes [80] and in healthy subjects after a 7-day high-fructose diet [81]. Adipose tissue lipolysis is primarily suppressed by insulin [82], but fructose ingestion does not stimulate a strong insulin excursion [43]. Another as-yet-unknown signal likely mediates this physiological effect of fructose ingestion.

Obesity, inflammation, and other mechanisms promote resistance to insulin’s antilipolytic effect [83]. This phenomenon is commonly referred to as adipose tissue insulin resistance and contributes to the increased release of FA from fat depots [84], which may contribute to intrahepatic lipid accumulation and the pathogenesis of NAFLD [85]. In obese insulin-resistant subjects, a relatively small increase in insulin following a fructose-rich meal or drink may not be sufficient to suppress adipose tissue lipolysis [86]. This would result in the continuous mobilization of endogenous energy stores under conditions of sufficient nutrient (ingested fructose) availability. These calories may then be diverted to the liver.

Increased visceral adiposity is strongly associated with adipocyte insulin resistance [87]. Since visceral adipose tissue releases FA into the portal circulation, which directly drains to the liver, this adipose compartment is particularly important in the context of NAFLD [26]. In one 10-week diet-intervention trial, the consumption of fructose, but not glucose, promoted the accumulation of visceral adipose tissue [55]. At the same time, 24-h average plasma FA levels were unchanged after the fructose diet, and the authors conclude that the harmful effects of fructose are not likely due to an effect on FA mobilization. However, since visceral lipolysis and/or portal FA levels were not quantified, these data do not exclude the possibility that chronic high fructose consumption contributes to an increased adipose tissue-liver FA flux.

There is some indirect evidence in support of this possibility. Insulin signaling in visceral white adipose tissue of rats is attenuated after 2 months of fructose, but not glucose, supplementation [88]. In another rat study, the administration of a high-fructose diet increased FA release from isolated adipocytes, as compared to glucose [89]. Human observational studies link the increased consumption of sugar-sweetened (fructose-containing) beverages to the accumulation of visceral adipose tissue [90] as well as intrahepatic lipids [91]. In addition, the consumption of excess calories from fructose contributes to weight gain [92], which promotes visceral adiposity, inflammation, and insulin resistance [83]. Thus, it remains debated whether fructose consumption directly influences adipocyte lipolysis and/or visceral adiposity, but it may contribute to an increase in adipose-to-liver FA trafficking through several indirect mechanisms.

### 3.3. Fructose and Hepatic Lipoprotein-Triglyceride Uptake

Approximately 15% of intrahepatic lipids are derived from dietary fat [18], and specific types of dietary fat, such as saturated FA [93], may increase intrahepatic lipid accumulation. Triglycerides from chylomicrons are hydrolyzed by lipoprotein lipase (LPL) for storage or oxidation in peripheral tissues. Spillover of FA from lipolysis of chylomicron-triglycerides is one route by which dietary lipids may end up in the liver [94]. Another is the clearance of chylomicron remnants, which carry triglycerides in addition to cholesteryl esters, after binding to the low-density lipoprotein (LDL) receptor on hepatocytes [95,96].

Hepatic lipoprotein metabolism also involves the secretion of VLDL particles (discussed below), remodeling of VLDL remnants, and clearance of cholesterol-rich LDL particles through LDL receptor-mediated endocytosis (for reviews, see [94,97]). This last route may also contribute, albeit limitedly, to hepatic triglyceride import. However, the hypertriglyceridemia associated with visceral adiposity is primarily due to impaired clearance of VLDL-triglycerides and lipoprotein remnants [94,97], suggesting that lipoprotein-triglyceride uptake is not necessarily increased in the context of the metabolic syndrome. In fact, tracer studies of triglyceride kinetics in obese humans suggest that liver fat is a determinant of lipoprotein synthesis, but not of lipoprotein clearance [50].

Short-to-medium-term high-fructose diets (conditionally) raise VLDL-associated plasma triglycerides in humans [55,98,99]. It is therefore likely that such diets also increase VLDL remnant concentrations and, by extension, hepatic remnant uptake [100]. We are, however, not aware of studies that have directly investigated the effect of an acute fructose load or chronic fructose consumption on hepatic lipoprotein (remnant) uptake in humans. In hamsters, high-fructose feeding was recently associated with the development of dyslipidemia and increased PCSK9-mediated hepatic LDL receptor degradation [101]. This suggests that decreased hepatic lipoprotein-triglyceride uptake may be one mechanism by which fructose contributes to dyslipidemia. However, the effect of fructose on hepatic lipoprotein handling in the context of NAFLD needs further exploration.

### 3.4. Fructose and β-Oxidation

Fructose is a high-energy nutrient. Its triose phosphate metabolites can enter the citric acid cycle for intrahepatic oxidation or the synthesis of glucose, lactate, and/or lipids (Figure 1). Most human cells lack the fructolysis machinery, but will readily use the newly synthesized glucose and lactate as energy source. Thus, via this two-step mechanism [102], fructose produces an energy source shift from lipid to carbohydrate oxidation [39,41], and this may influence the accumulation of intrahepatic lipids by decreasing hepatic lipid oxidation and output (Figure 2). This was demonstrated in rat livers, where perfusion with fructose-spiked blood inhibited hepatic β-oxidation of FA by substrate competition [103,104], as well as in healthy subjects, where the ingestion of fructose acutely increased carbohydrate oxidation and suppressed fat oxidation rates [79]. It is noteworthy that the inhibitory effect of fructose on FA oxidation in rat livers was amplified by co-administration of insulin [103], suggesting that it may be altered in the context of insulin resistance. Nevertheless, whether impaired β-oxidation is quantitatively relevant to clinical NAFLD remains inconclusive [26,48,105].

### 3.5. Fructose and Hepatic VLDL Secretion

Excess triglycerides are secreted from the liver in triglyceride-rich VLDL, which functions as a transporter for endogenous lipids in otherwise hydrophobic plasma. Most [49,50,51], but not all [19] of the available evidence indicates that the increased availability of liver fat in the context of NAFLD drives up hepatic VLDL secretion. Thus, in order for hepatic steatosis to progress, the rate of triglyceride synthesis (from DNL or FA re-esterification) must overcome the increased rate of VLDL secretion [105]. To our knowledge, it is unknown if fructose metabolites directly influence hepatic VLDL synthesis or secretion pathways. It is, however, generally thought that chronic fructose consumption and the increase in lipid synthesis will provide the liver with excess triglycerides, allowing for increased VLDL secretion [106].

Under postprandial conditions, insulin enhances LPL activity and triglyceride uptake in adipose tissue [106]. Fructose ingestion, however, does not induce high insulin levels and is, therefore, associated with relatively lower postprandial (insulin-stimulated) triglyceride clearance. This leads to large chylomicron and VLDL particles in circulation, hypertriglyceridemia, and, possibly, more remnants to circle back to the liver.

Finally, long-term exposure to intrahepatic lipid species results in endoplasmic reticulum (ER) and oxidative stress, which increases the degradation of apolipoprotein B100 and reduces VLDL secretion [107], suggesting that impaired hepatic triglyceride export may worsen hepatic steatosis in the long term. Accordingly, patients with NASH are characterized by distinctly impaired apolipoprotein B100, but not global protein, synthesis [52].

## 4. Does Fructose Consumption Cause Hepatic Steatosis in Humans?

### 4.1. Epidemiological Evidence

Several epidemiological studies have evaluated the association between average daily fructose intake and hepatic steatosis. In practice, human fructose consumption is largely driven by added sugars, and the intake of sugar-sweetened beverages generally corresponds to total fructose intake [108]. Data from the Framingham cohort indicated that the increased consumption of sugar-sweetened beverages is associated with fatty liver, independent of possible confounders such as overall energy intake [91]. In another study, patients with NAFLD consumed two-to-three times more fructose than matched controls [109]. Contrary, data from a large Finnish cohort did not support an association between fructose intake and NAFLD [110], and the NASH Clinical Research Network suggested that fructose intake is associated with reduced hepatic steatosis, but increased disease progression (that is, hepatic fibrosis) [111]. Thus, although there is some evidence linking real-world fructose consumption to the development of fatty liver, the epidemiological evidence is largely inconsistent and, as we stand, does not support definitive conclusions [112]. Moreover, dietary sugar intake has clearly been linked to weight gain [113], indicating that any association between fructose intake and NAFLD may be confounded by a positive energy balance and/or obesity.

### 4.2. Controlled Diet-Intervention Studies

Controlled diet-intervention trials prospectively compare the effects of a fructose-intervention diet to the effects of a control diet. As such, they are less susceptible to bias than epidemiological studies. These trials can be categorized as isocaloric, where the fructose-intervention diet and the control diet have the same energy content, or hypercaloric, where fructose supplementation provides extra energy relative to the control diet. Although short-term hypercaloric fructose-intervention trials may provide mechanistic insight into specific effects of fructose [114], they do not control for energy balance and their results cannot be easily generalized. Isocaloric fructose-intervention trials are most valuable to evaluate whether fructose, more than other nutrients, causes metabolic disease.

Two meta-analyses of data from four isocaloric fructose vs. glucose-intervention trials in a total of 63 healthy or overweight participants suggested that fructose, in and of itself, does not contribute to increased intrahepatic lipids during short-term (1–8 weeks) studies [112,115]. Both analyses did indicate that hypercaloric fructose, when compared to a weight-maintenance control diet, contributes to NAFLD, suggesting that excess calorie consumption, regardless of the source of these calories, induces liver fat accumulation. We are aware of two more isocaloric trials that have assessed the effects of fructose on liver fat since the synthesis of these meta-analyses. Schwarz et al. [116] compared high doses of fructose to isocaloric doses of complex carbohydrates during consecutive 9-day periods in a crossover design. All eight participants had higher rates of DNL and higher hepatic fat content after the fructose diets. Agebratt et al. [117] compared 2-month high-fruit vs. high-nut diets in 30 healthy participants. Here, the 3-fold increase in fructose intake in the fruit group was not associated with a change in liver fat.

Thus, so far, only one of the six available isocaloric comparisons supports the hypothesis that fructose, in and of itself, has adverse effects on clinically meaningful outcomes of NAFLD. It should, however, be noted that these observations are limited by the small sample sizes and short durations of the available trials. We are in clear need of large, long-term trials to determine if real-world fructose exposure contributes to the development of NAFLD and other metabolic diseases. In addition, fructose-containing sugars are an important source of excess calories, suggesting that a reduction of their intake may still have potential for the prevention of NAFLD [114].

### 4.3. Fructose Reduction as a Therapeutic Strategy

Current management of NAFLD primarily involves diet and lifestyle recommendations. Both caloric restriction and physical activity effectively reduce hepatic steatosis, but most patients are unable to stick with such lifestyles in the long term [118]. Targeting fructose-associated hepatic DNL may offer an alternative strategy to the management of NAFLD. Already, rodents can be protected from fructose-induced hepatic fat accumulation by several molecular interventions, including liver-specific activation of AMP-activated protein kinase [119] or activation of glutamate dehydrogenase, which promotes a metabolic shift from lipogenesis and gluconeogenesis toward glutamate synthesis [120]. Specific antilipogenic agents are not currently available for humans, but a better understanding of fructose metabolism will undoubtedly facilitate the development of novel pharmacological therapies. In addition, if fructose consumption plays an important role in the development and progression of NAFLD [121], then targeted low-fructose lifestyle interventions may have a beneficial effect on liver fat; but is there evidence for this?

A reduction in fructose intake can be achieved by several means, including low-carbohydrate diets, low-sugar diets, and/or the substitution of dietary fructose with low-fructose sweeteners. Volynets et al. [122] assessed the effects of a 6-month dietary intervention that focused on a reduction of fructose intake (~50% of baseline) in 10 subjects with NAFLD. Liver fat reduced significantly, but, as this was an uncontrolled trial, it cannot be ruled out that this was driven by a reduction in total energy intake and associated weight loss. Schwartz et al. [123] recently provided 41 obese children with metabolic syndrome features with isocaloric, but fructose-restricted (~33% of baseline) meals for 9 days. Both fractional DNL and liver fat were markedly lower after 9 days of fructose restriction. Although this was an uncontrolled trial as well, the dietary intervention was isocaloric and weight loss was minimal, suggesting that short-term fructose restriction reduces DNL and liver fat in obese children. We are not aware of any trials that have evaluated the effects of fructose reduction on liver fat in NAFLD in a controlled study design. In one 6-month pilot study, children with NAFLD tended to have lower plasma levels of liver enzymes with diet education aimed at reducing fructose intake [124], but this finding has yet to be replicated in a larger sample.

Finally, a recent meta-analysis of four trials indicated that the consumption of a low-carbohydrate diet is associated with improved liver fat content in subjects with NAFLD [125]. In two of the included trials, the low-carbohydrate/high-fat diet was adequately compared to a control diet, suggesting that low-carbohydrate diets may be more effective for liver fat reduction than low-fat diets. It remains to be determined if a reduction in fructose intake in the context of a low-carbohydrate diet is responsible for part of these results.

## 5. Does Fructose Consumption Cause Disease Progression in Humans?

### 5.1. Fructose and Hepatic Insulin Resistance

Hepatic lipid accumulation in the context of NAFLD is often associated with hepatic insulin resistance [126,127,128,129,130,131,132,133,134], and NAFLD is considered an important risk factor for type 2 diabetes [11]. Emerging evidence points to the accumulation of specific intrahepatic lipid species, most importantly diacylglycerol (DAG), as a cause of lipid-mediated insulin resistance. Increased hepatic DAG content activates protein kinase Cε (PKCε) in genetic or diet-induced obese animal models [135,136,137]. This results in its translocation from the cytosol to the cell membrane, where it phosphorylates insulin receptor threonine^1160^ and inhibits insulin receptor kinase activity/downstream signaling [138]. We have recently demonstrated that obese humans with hepatic insulin resistance are also characterized by intrahepatic DAG accumulation and PKCε activation/translocation [139], supporting the translational relevance of this mechanism of hepatic insulin resistance.

There is also preclinical evidence to suggest that fructose itself promotes hepatic DAG accumulation. Short-term high-fructose feeding to rodents increased hepatic DAG content [140,141,142] and was associated with PKCε activation and impaired insulin signaling [78]. In addition, fructose activates ChREBP and promotes hepatic gluconeogenesis independently of hepatic insulin signaling, which may contribute to hepatic insulin resistance (Figure 3) [58]. In support of these mechanisms linking fructose to hepatic insulin resistance, data from our recent systematic review and meta-analysis of diet-intervention trials in nondiabetic humans demonstrate that fructose consumption has more harmful effects on hepatic insulin sensitivity than the isocaloric consumption of glucose or other carbohydrates [25].

### 5.2. Fructose and NASH

Fructose metabolism has been suggested to induce hepatotoxic changes that contribute to disease severity in the context of NAFLD. Epidemiological studies have linked increased daily fructose intake to NASH in children and adolescents [143] as well as to increased hepatic fibrosis in adults [111]. It has been suggested that uric acid mediates some of the hepatotoxic effects of fructose. Ingested fructose is phosphorylated by hepatic KHK to produce fructose-1-phosphate (Figure 1). This reaction rapidly depletes intrahepatic adenosine triphosphate (ATP) levels and promotes adenosine monophosphate (AMP) production [144]. Elevated levels of AMP stimulate the production of uric acid, which is the final product of purine metabolism [145]. Uric acid, in turn, has been shown to induce intracellular oxidative stress [146], which is considered an important driver of progression to NASH [147]. Circulating uric acid levels are independently associated with NASH [143].

Short-term fructose-intervention trials are not designed to evaluate the effect of fructose on NASH directly, but some of these trials have studied the effect of fructose on serum uric acid levels. In one meta-analysis, short-term hypercaloric fructose supplementation increased circulating levels of uric acid [148]. Accordingly, daily fructose intake is predictive of uric acids levels in epidemiology [143], and a low-fructose diet was associated with a decrease in uric acid in overweight or obese individuals [149]. 

We also point out that hyperuricemia increases the risk for gout [150], the metabolic syndrome [151], and, possibly, cardiovascular mortality [152]. It may also contribute to insulin resistance by hindering endothelial function [153,154]. Consequently, while direct evidence linking fructose ingestion to increased liver fat in humans remains inconclusive, several lines of evidence, including preclinical, epidemiological, and interventional, support effects of fructose consumption on the development of hepatic insulin resistance, hyperuricemia, and NASH. This further underlines the potential of fructose avoidance for the prevention of NAFLD-associated diseases.

## 6. Conclusions

The scientific literature regarding the role of fructose consumption in the development of hepatic steatosis may seem somewhat inconsistent. On one hand, the available evidence from human epidemiological and interventional studies does not support the hypothesis that fructose, when consumed in isocaloric amounts, causes more liver fat accumulation than other energy-dense nutrients. The observed prosteatotic effect of fructose in hypercaloric trials [115] is likely confounded by associated weight gain [92], and this level of evidence is insufficient to adequately guide the public and policy makers on the use of dietary fructose vs. other products.

On the other hand, observations from numerous mechanistic studies in cells, animals, healthy volunteers, and patients clearly demonstrate that fructose has lipogenic potential. Hepatic fructose metabolism rapidly produces gluconeogenesis and lipogenesis precursors, while intermediary fructose metabolites also act as nutritional regulators of the major transcription factors that control these pathways. Accordingly, fructose ingestion increases plasma triglyceride levels [43,44,45,46,47], and high-fructose diets promote hepatic DNL [46,54,55]. Hepatic DNL is an important contributor to intrahepatic lipids in human NAFLD [18,19,20], suggesting that long-term fructose overconsumption may promote mechanisms that drive NAFLD development. Moreover, the overall evidence linking increased fructose intake to hepatic insulin resistance, increased uric acid concentrations, and NAFLD severity/progression is more convincing.

Finally, we are confident that a deeper understanding of the cellular mechanisms governing hepatic fructose metabolism will reveal novel targets for NAFLD/NASH, hypertriglyceridemia, and hepatic insulin resistance/type 2 diabetes.

## Appendix A. Literature Search and Selection Strategy

We searched for relevant peer-reviewed English-language publications in the MEDLINE (www.ncbi.nlm.nih.gov/pubmed), EMBASE (ovidsp.tx.ovid.com), and Cochrane Library (www.cochranelibrary.com) databases from inception through 26 June 2017. The literature search was focused on fructose, lipogenesis, NAFLD, NASH, and related terms. We manually reviewed reference lists of selected articles for additional publications. Entries identified through the search were screened for relevance, and full-text articles were retrieved for in-depth review and methodological quality assessment.

## Appendix B. Recommended Further Reading

Mosca et al. [155] discussed the roll of soft drink consumption in pediatric metabolic disease. Rippe and Angelopoulos [156] evaluated recent observations from randomized controlled trials and meta-analyses into the effects of sugar consumption on important metabolic outcomes and health-related issues. Alwahsh and Gebhardt [157] approached the issue from different angles and highlight how fructose consumption may be related to inflammation, gut microbiota, and central nervous system mechanisms. Finally, Kahn and Sievenpiper [158] take the counterpoint stand and argue that there is no convincing evidence to implicate sugar in the development of obesity and its related diseases.
